# Alterations in DNA methylation associate with reduced migraine and headache days after medication withdrawal treatment in chronic migraine patients: a longitudinal study

**DOI:** 10.1186/s13148-023-01604-8

**Published:** 2023-12-12

**Authors:** Divya Mehta, Irene de Boer, Heidi G. Sutherland, Judith A. Pijpers, Charlene Bron, Charlotte Bainomugisa, Larisa M. Haupt, Arn M. J. M. van den Maagdenberg, Lyn R. Griffiths, Dale R. Nyholt, Gisela M. Terwindt

**Affiliations:** 1 Centre for Genomics and Personalised Health, Queensland University of Technology, 60 Musk Avenue, Brisbane, QLD 4059 Australia; 2 Centre for Data Science, Queensland University of Technology, 2 George Street, Brisbane, QLD 4000 Australia; 3 School of Biomedical Sciences, Faculty of Health, Queensland University of Technology, 2 George Street, Brisbane, QLD 4000 Australia; 4 Department of Neurology, Leiden University Medical Center, Albinusdreef 2, PO Box 9600, 2300 RC Leiden, The Netherlands; 5 Department of Human Genetics, Leiden University Medical Center, Albinusdreef 2, PO Box 9600, 2300 RC Leiden, The Netherlands

**Keywords:** DNA methylation, Migraine, Chronic migraine, Epigenome-wide association study, EWAS, Longitudinal, Medication overuse headache, Acute medication withdrawal, Treatment response, *HDAC4*, *MARK3*

## Abstract

**Background:**

Chronic migraine, a highly disabling migraine subtype, affects nearly 2% of the general population. Understanding migraine chronification is vital for developing better treatment and prevention strategies. An important factor in the chronification of migraine is the overuse of acute headache medication. However, the mechanisms behind the transformation of episodic migraine to chronic migraine and vice versa have not yet been elucidated. We performed a longitudinal epigenome-wide association study to identify DNA methylation (DNAm) changes associated with treatment response in patients with chronic migraine and medication overuse as part of the Chronification and Reversibility of Migraine clinical trial. Blood was taken from patients with chronic migraine (*n* = 98) at baseline and after a 12-week medication withdrawal period. Treatment responders, patients with ≥ 50% reduction in monthly headache days (MHD), were compared with non-responders to identify DNAm changes associated with treatment response. Similarly, patients with ≥ 50% versus < 50% reduction in monthly migraine days (MMD) were compared.

**Results:**

At the epigenome-wide significant level (*p* < 9.42 × 10^–8^), a longitudinal reduction in DNAm at an intronic CpG site (cg14377273) within the *HDAC4* gene was associated with MHD response following the withdrawal of acute medication. HDAC4 is highly expressed in the brain, plays a major role in synaptic plasticity, and modulates the expression and release of several neuroinflammation markers which have been implicated in migraine pathophysiology. Investigating whether baseline DNAm associated with treatment response, we identified lower baseline DNAm at a CpG site (cg15205829) within *MARK3* that was significantly associated with MMD response at 12 weeks.

**Conclusions:**

Our findings of a longitudinal reduction in *HDAC4* DNAm status associated with treatment response and baseline *MARK3* DNAm status as an early biomarker for treatment response, provide support for a role of pathways related to chromatin structure and synaptic plasticity in headache chronification and introduce *HDAC4* and *MARK3* as novel therapeutic targets.

**Supplementary Information:**

The online version contains supplementary material available at 10.1186/s13148-023-01604-8.

## Introduction

Chronic migraine is a highly disabling migraine subtype affecting nearly 2% of the general population [1–3]. It is defined by the occurrence of headaches on ≥ 15 days/month for > 3 months, of which ≥ 8 days fulfil migraine criteria [2]. The majority of patients overuse acute headache medications including analgesics, triptans, and/or opioids, which is an important factor in the maintenance of chronic migraine [1, 3, 4]. Medication withdrawal therapy is effective in reducing headache frequency in 60% of patients with chronic migraine [4].

The mechanisms behind the transformation of episodic migraine to chronic migraine and vice versa have not yet been elucidated. It has been suggested that epigenetic modifications may be important in this transition [5]. Epigenetic modifications encompass a variety of chemical and structural changes to chromosomal regions with effects of genetic and environmental factors on local DNA transcription. DNA methylation (DNAm) is an important epigenetic modification that involves the covalent binding of methyl groups to CpG (5′-cytosine-phosphate-guanine-3′) sites that are distributed throughout the genome [6]. DNAm status is dynamic but can be inherited from parent to daughter cells. As such, DNAm changes can lead to short- and long-lasting changes influencing disease [6], with its dynamic nature enabling these changes to be reversible. The hypothesis that epigenetic changes are implicated in migraine may suggest that altered synaptic plasticity of neurons is accompanied by changes in the epigenome leading to the maintenance of chronification of migraine. If treatment results in conversion back to episodic migraine, these epigenetic processes might also be reverted. Moreover, as some patients do not respond to withdrawal and/or prophylactic therapy, such as monoclonal CGRP-antibodies [4, 7], it is important to understand this clinical variation.

Epigenetic factors have been previously implicated in migraine pathophysiology. For example, one of the migraine genome-wide association study (GWAS) loci, the *PRDM16* locus, encodes a histone H3 lysine methyltransferase that acts as a transcriptional regulator [8, 9]. Moreover, in rats, inducing cortical spreading depolarisation (CSD), the underlying mechanism of migraine aura, caused changes in chromatin, including histone H3 lysine methylation, at neuroprotective gene loci and retrotransposon elements [10, 11]. Furthermore, depression/anxiety, obesity, stress, and female sex hormones have all been implicated in migraine pathophysiology, and are known to exert their physiological effects partly through epigenetic mechanisms [11–15]. Some epigenome-wide association studies (EWAS) have indicated suggestive differences in DNAm profiles between patients with migraine and controls, with possible involvement in chronification [5, 16, 17], although with limited sample sizes no specific loci were significant. A recent study by Carlsen et al. (2023) examined DNAm in 120 patients diagnosed with medication overuse headache (some with migraine) and found significant differences in immune cell proportions, as well as differentially methylated CpGs localised to three genes (*CORIN*, *CCKBR*, and *CLDN9*) between MOH and controls (which included both episodic migraine and healthy controls) [18]. However, the Carlsen et al. (2023) study comprised heterogeneous treatment groups, did not examine chronic migraine patients, and found no CpGs associated with reduced headache or migraine days.

We previously conducted the Chronification and Reversibility of Migraine (CHARM) clinical trial study at Leiden Headache Center, which assessed efficacy of acute medication withdrawal and add-on therapy with botulinum toxin A (botox) for chronic migraine and medication overuse [4]. While withdrawal treatment significantly reduced monthly headache and migraine days in responders, botox did not show any additional benefit. Hypothesising that a reduction in headache and migraine days would be accompanied by specific changes in DNAm, here we utilised our unique CHARM clinical trial resource and performed a novel study to examine longitudinal epigenome-wide DNAm profiles from peripheral blood of patients with chronic migraine before and after medication withdrawal treatment. Furthermore, we determined whether DNAm status at baseline was indicative of treatment response. Our findings identify specific epigenetic changes related to favourable treatment response. Understanding these underlying biological processes will ultimately help identify new treatment targets and preventive opportunities.

## Results

### Demographics and treatment efficacy

**Fig. 1:**
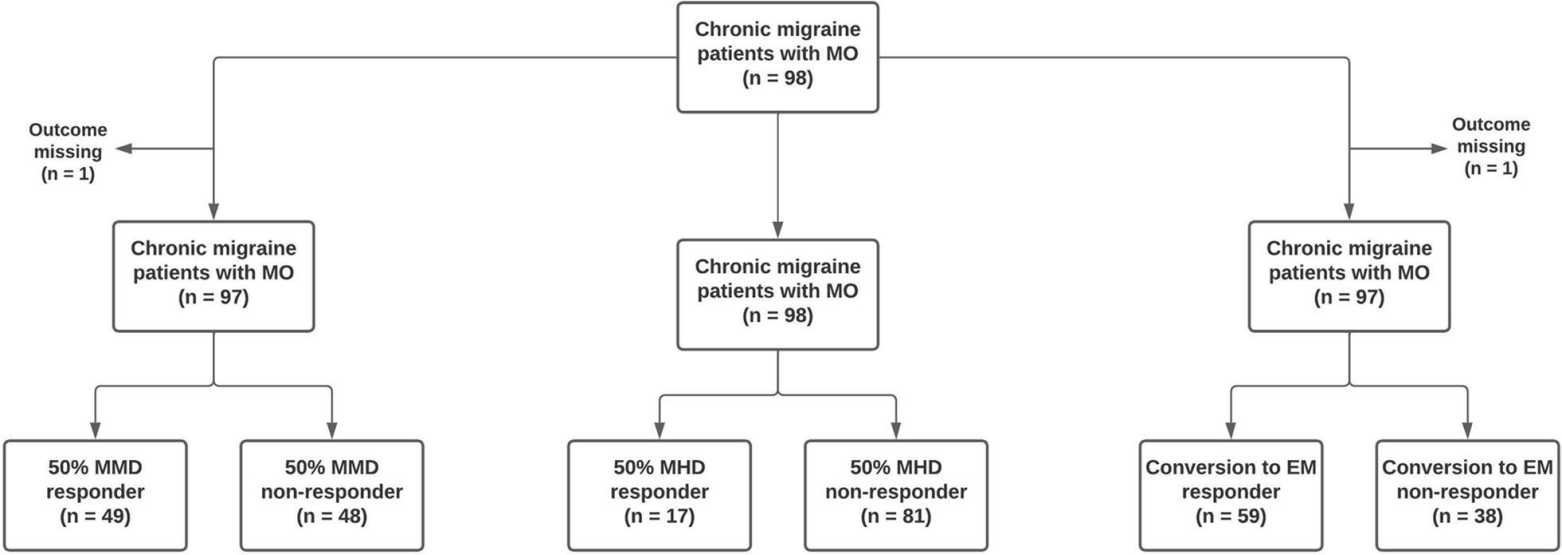
Flow chart treatment effect. MO = medication overuse, MHD = monthly headache days, MMD = monthly migraine days, EM = episodic migraine

Of the 98 chronic migraine patients with medication overuse (simple analgesics and/or triptans) the average age was 47.0 (standard deviation [SD] ± 10.1). At baseline (*T* = 0) most patients used no prophylactic medication (65.3%). Of the participants who were rapidly tapering off their medication at *T* = 0, patients were using beta blockers (*n* = 7), valproate (*n* = 6), topiramate (*n* = 3), pizotifen (*n* = 2), candesartan (*n* = 7) or a combination or other type of prophylactics (*n* = 9). Characteristics of the cohort are shown in Table 1. The 50% responder rate (% individuals with ≥ 50% reduction from baseline) was 16.3% for MHD and 49.5% for MMD (Fig. 1). Of all patients, 59.8% converted back to episodic migraine by the end of the 12-week withdrawal period (*T* = 1). In the CHARM study we previously demonstrated that in patients with chronic migraine and medication overuse, botox did not lead to any additional benefits over acute withdrawal alone [4]. As expected, this also applied to the current sample (50% responder rate: MMD 51.9% in the placebo group versus 47.8% in the botox group; 50% responder rate MHD: 19.2% in the placebo group versus 15.2% in the botox group; and rate conversion back to episodic migraine: 55.8% in the placebo group versus 65.2% in the botox group). Finally, as to be expected after randomisation, patients receiving botox or placebo were not significantly different in age, gender, BMI, and MHD, MMD, and monthly medication days at baseline (*p* > 0.05).

**Table 1:** Patient demographics at baseline

Characteristic	Value
Total, *n*	98
Female, *n* (%)	74 (75.5)
Age, mean ± SD	47.0 ± 10.1
BMI, mean ± SD	25.4 ± 4.6
MMD, *n* ± SD	15.2 ± 5.4
MHD, *n* ± SD	21.2 ± 4.8

BMI, body mass index; MMD, monthly migraine days; MHD, monthly headache days; SD, standard deviation

### Longitudinal epigenome-wide DNA methylation differences after treatment

**Fig. 2:**
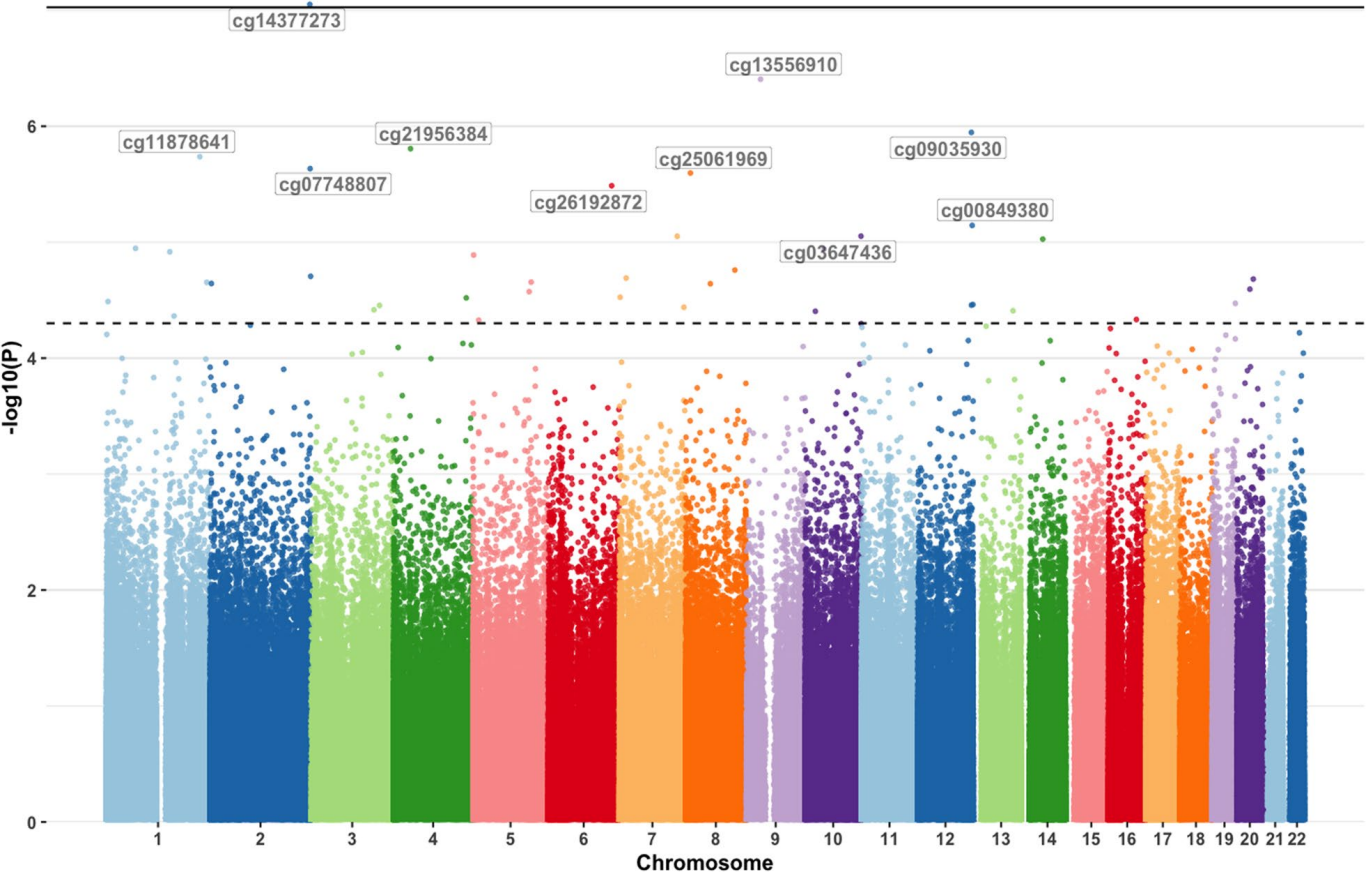
Manhattan plot from the epigenome‐wide association study. Manhattan plot showing the –log_10_(*p* value) for each CpG site associated with changes in DNA methylation in monthly headache days (MHD) responders versus non-responders. The threshold for epigenome-wide significant association (*p* < 9.42 × 10^–8^) is indicated by a solid black line. The threshold for epigenome-wide suggestive association (*p* < 5 × 10^–5^) is indicated by a dashed black line

**Fig. 3:**
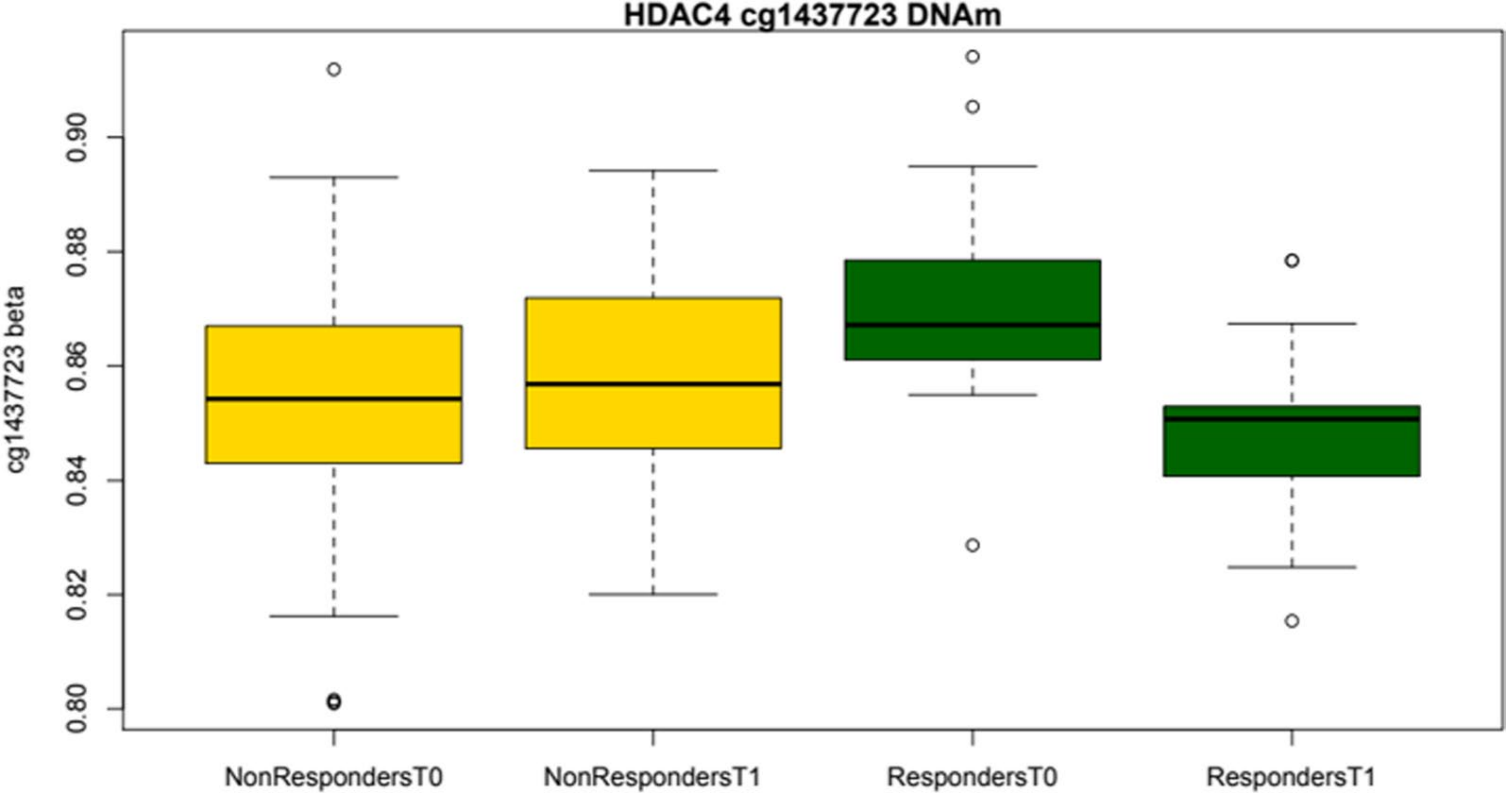
Median of differential *HDAC4* DNA methylation in patients with and without ≥ 50% reduction in monthly headache days (MHD)

We investigated DNAm levels at two timepoints (baseline [*T* = 0] and after treatment [*T* = 1]) to identify DNAm differences in CpG sites that were associated with treatment response via linear mixed effects models. The Q-Q plot (Additional file 1: Fig. S1) from the epigenome‐wide association analysis of changes in DNAm in MHD responders versus non-responders indicated no systematic technical bias or inflation of the test results. The Q-Q plot for MMD produced analogous results (data not shown). At the stringent epigenome-wide *significant* level (*p* < 9.42 × 10^–8^), changes in DNAm at an intronic CpG site (probe cg14377273) within the histone deacetylase 4 (*HDAC4*) gene was associated with MHD response following the withdrawal of acute medication (*p* = 8.9 × 10^–8^, Table 2, Figs. 2, 3). Post-hoc sensitivity analyses showed that this CpG site remained significant even after adjusting for prophylactics use and medication overuse type (*p* = 9.4 × 10^–8^). Another 40 CpGs in *HDAC4* were nominally associated with MHD response (0.05 < *p* < 3.18 × 10^–4^, Additional file 2: Table S1). Using the Blood Brain DNA methylation comparison tool, we observed that several of the *HDAC4* CpG probes had a high correlation between blood and brain prefrontal cortex DNAm patterns (e.g., cg15376007: *r* = 0.47, *p* = 2.47 × 10^–5^; and cg10118705: *r* = 0.26, *p* = 0.028). Functional annotation using the Drug-Gene Interaction database (DGIdb) indicated that *HDAC4* is a ‘clinically actionable’ gene with a range of HDAC inhibitors available [19]. At the epigenome-wide *suggestive* level (*p* < 5 × 10^–5^), an additional 40 CpGs across the genome were associated with MHD response (4.70 × 10^–5^ < *p* < 3.93 × 10^–7^, Additional file 2: Table S2). Overall, the CpGs associated with MHD response (*p* < 0.05) were significantly enriched for genes previously associated with migraine and/or headache traits in GWAS (*p* value of enrichment = 0.011), indicating that the overlap of genes was more than expected by chance [20–24]. Although no CpG sites showed epigenome-wide significant association with MMD response, seven CpGs were associated at the suggestive level (4.03 × 10^–5^ < *p* < 2.30 × 10^–6^, Additional file 2: Table S3). Similarly, no CpG sites showed epigenome-wide significant association when comparing the converted episodic migraine individuals to the remaining chronic migraine individuals, however, ten CpGs were associated at the suggestive level (4.86 × 10^–5^ < *p* < 1.04 × 10^–5^, Additional file 2: Table S4).

**Table 2:** CpG sites significantly associated with treatment efficacy in chronic migraine

CpG	Gene	Chr	Location in gene	Effect size [SD]	*P* value	Direction (T0 to T1)	Analysis Model
cg14377273	*HDAC4*	2	Body	0.02 [0.016]	8.9 × 10^–8^	reduced DNAm	longitudinal DNAm ~ MHD
cg15205829	*MARK3*	14	Body	0.02 [0.001]	4.1 × 10^–8^	*not applicable*	baseline DNAm ~ MMD

CpG, 5′-C-phosphate-G-3′; Chr, chromosome; SD, standard deviation; MHD, monthly headache days; MMD; monthly migraine days

### Associations between epigenome-wide DNA methylation at baseline and treatment response

**Fig. 4:**
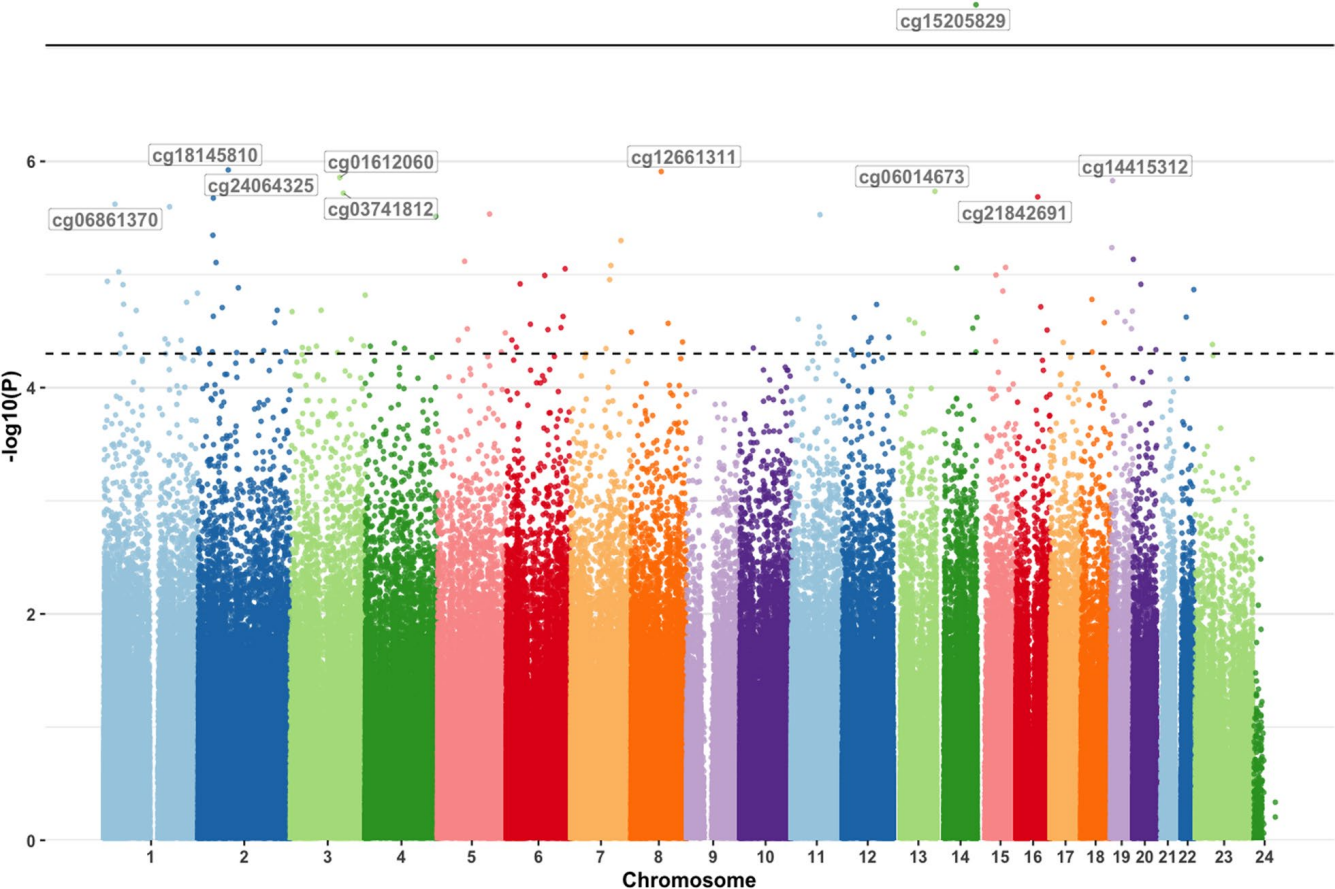
Manhattan plot from the epigenome‐wide association study of baseline DNA methylation predicting treatment response. Manhattan plot showing the –log_10_(*p* value) for each CpG site associated with baseline DNA methylation in responders versus non-responders at T1. The threshold for epigenome-wide significant association (*p* < 9.42 × 10^–8^) is indicated by a solid black line. The threshold for epigenome-wide suggestive association (*p* < 5 × 10^–5^) is indicated by a dashed black line

Next, we assessed whether DNAm profiles at baseline associated with treatment response after 12 weeks. Analogous to the longitudinal DNAm results, Q-Q plots of the baseline DNAm analysis for MHD and MMD indicated no systematic technical bias or inflation of the test results (data not shown). Methylation levels at one CpG probe (cg15205829) within the *MARK3* gene was associated with MMD response (*p* = 4.13 × 10^–8^) at the epigenome-wide significant threshold, with decreased DNAm levels at baseline associated with reduced MMD (Table 2, Fig. 4). At the epigenome-wide suggestive level, an additional 114 CpGs across the genome were associated with MMD response, including a second probe (cg26267011, *p* = 4.84 × 10^–5^) in *MARK3* (4.98 × 10^–5^ < *p* < 1.19 × 10^–6^, Additional file 2: Table S5, Fig. 4). *MARK3* is currently not listed as a ‘clinically actionable’ drug target in DGIdb (i.e., there are no known drug-gene associations for *MARK3*). No CpG sites were associated with MHD response at the epigenome-wide significant threshold; however, 109 CpGs were associated at the suggestive level (4.82 × 10^–5^ < *p* < 6.42 × 10^–7^, Additional file 2: Table S6).

## Discussion

In this large longitudinal epigenome-wide association study (EWAS) we identified DNAm differences significantly associated with response to medication withdrawal treatment in individuals with chronic migraine. Our findings of CpG sites associated with improvement of headache and migraine days after treatment are of important clinical relevance. A longitudinal change in DNAm at a CpG site within an intron of *HDAC4* was associated with MHD response, while baseline DNAm levels at a CpG in *MARK3* were associated with MMD response at 12 weeks.

The strongest association with reduced headache days was for an intronic CpG within *HDAC4* (cg14377273, *p* = 8.90 × 10^–8^); post-hoc sex-specific analysis indicated this CpG was associated in both sexes (*p* = 3.73 × 10^–6^ in females and *p* = 0.005 in males). Nearby SNP rs7581200 is an expression quantitative trait locus (eQTL) for *HDAC4* in the GTEx database suggesting localisation of a regulatory element in this region. Another 40 CpG probes spanning the *HDAC4* gene showed associations at the nominal level (Table S1). *HDAC4* encodes a Class IIa histone deacetylase, and along with co-repressors such as MEF2D encoded by a previously reported migraine GWAS locus [20, 23], its activity targets lysine residues on core histone tails to repress transcription. HDAC4 is highly expressed in the brain and is a major player in synaptic plasticity [25, 26] and modulates the expression and release of several neuroinflammation markers, including HMGB1 and NF-κB [27, 28], which have been implicated in migraine pathophysiology [29]. HDAC4 has previously been implicated in chronic pain disorders such as fibromyalgia [30, 31]. Furthermore, selective knockout of *Hdac4* in peripheral sensory neurons in two independent mice lines found attenuated development of chronic inflammatory pain, indicating a role for HDAC4 in peripheral sensitization and inflammation-associated thermal hypersensitivity [32]. The conditional *Hdac4* deletions resulted in significant transcriptional dysregulation of genes involved in pain sensitivity, such as *Calca* and *Trpv1*, encoding Calcitonin Gene-Related Peptide 1 (CGRP) and Transient Receptor Potential Cation Channel Subfamily V Member 1 (TRPV1) respectively—both also known to be involved in migraine [33, 34].

Differentially methylated CpGs were also detected in the genes *HDAC1* and *HDAC3* among sites with nominal association (*p* < 0.05) with reduced headache and migraine days in our study. There is evidence for several HDACs being involved in regulating the processes involved in pain chronification. For example, inhibition of HDAC6 decreased cephalic allodynia and reversed cyto-architectural changes in headache-processing brain regions in a mouse model of chronic migraine-associated pain [35]. Thus, non-specific HDAC inhibitors may present a therapeutic avenue for migraine and headache-related disorders via multiple pathways including regulating gene transcription as well as regulating structural proteins. Notably, valproate, a widely prescribed migraine prophylaxis, is a known inhibitor of HDAC activity [19]. Further support for the potential therapeutic utility of HDAC inhibitors in prevention and/or reversal of chronic migraine is provided by a study in a rat model of MOH, that found two pan-HDAC inhibitors (panobinostat and givinostat) reduced expression of the genes coding for calcitonin gene-related peptide (CGRP) and its receptor subunit Receptor Activity Modifying Protein 1 (RAMP1), whose proteins are known to have key roles in migraine pathogenesis and MOH [36].

The CpG predictive of favourable MMD response after medication withdrawal treatment at baseline was located in *MARK3*, which encodes microtubule affinity regulating kinase 3 (MARK3, also known as CTAK1). MARKs are serine/threonine kinases that regulate numerous cellular functions such as cell polarity, cell cycle progression, glucose metabolism and cytoskeletal dynamics [37, 38]. MARKs, including MARK3, regulate TRESK (TWIK-related spinal cord K^+^ channel, *KCNK18*) a major background K( +) channel of sensory neurons [39], in which mutations can lead to hyperexcitability of trigeminal ganglion neurons [40]. *MARK3* mRNA is specifically transported to and translated in axons of adult dorsal root ganglion neurons [41], raising the possibility that microtubule dynamics is coupled to the regulation of excitability in neurons [39]. Notably, downstream substrates of MARK3 include several HDACs, of which HDAC4 is one; and in response to specific signals, MARK3 phosphorylates HDACs on their binding sites for the adaptor protein 14–3–3, impairing interactions with 14–3–3, which regulates HDAC nuclear/cytoplasmic localisation and the transcriptional repressor function of HDACs [42]. Therefore, the potential role of MARK3 in migraine chronification may also be via regulation of HDACs.

The female sex hormone estrogen plays important roles in migraine frequency [43], synaptic plasticity [44], and in the regulation and recruitment of HDACs [45, 46]. With respect to HDAC4, Maddox et al. (2018) identified an estrogen-dependent association of *HDAC4* methylation and expression with fear regulation and PTSD risk in both female mice and women, which may contribute to the increased risk among women for PTSD [47]. Therefore, a pathway involving HDAC4 and estrogen might also partially explain why women have a higher risk for migraine chronification.

Our findings thus provide support for a potential role of pathways related to chromatin structure, and synaptic plasticity that may have relevance to migraine chronification and its reversibility. It remains to be determined whether such changes are a cause or consequence of a decreased frequency of attacks. Interestingly, a previous case–control EWAS comparing 36 female chronic headache patients to 35 female episodic headache patients found the two strongest associated loci were linked to brain-expressed genes (*SH2D5* and *NPTX2*) that are involved in the regulation of synaptic plasticity [5], although these were not experiment-wide significant. In contrast to our study, longitudinal analysis of MOH patients by Carlsen et al. (2023) did not find any significant DNAm changes associated with reduction in headache frequency over a 6-month period [18]. Importantly, our study was specifically restricted to chronic migraine patients undergoing medication withdrawal, while the Carlsen et al. (2023) study comprised a variety of migraine and tension headache patients, as well as heterogeneous treatment strategies.

We found little overlap between loci at the suggestive level of association between the different endpoints (i.e., MHD response, MMD response, or chronic to episodic migraine conversion). While this could be due to insufficient statistical power (to detect smaller effects), it suggests that while we might expect similarities between migraine and non-migraine days in patients with chronic migraine from a pathophysiological viewpoint, these days may be influenced by different mechanisms. For example, headache days in patients with chronic migraine with medication overuse might be due to medication overuse mechanisms as opposed to migraine-specific mechanisms. Enrichment analysis of CpGs associated with MHD response (*p* < 0.05) showed significant overlap with genetic loci previously associated with migraine and/or headache traits in GWAS (*p* = 0.011). Although, given that our study only assessed patients with chronic migraine and evaluated treatment, whereas GWAS tests for genetic differences between patients and controls, it is perhaps reasonable to expect only a modest overlap between genes associated with a reduction in MHD and GWAS loci.

Our study had several potential limitations. Firstly, before trial commencement, individuals were on different prophylactic medications, which may influence their epigenetic profile. However, 65% of patients were not using prophylactics at the beginning of the study and the remaining patients were tapered off medications as quickly as possible. Furthermore, we adjusted for prophylactic use in our analyses. Secondly, tissue-specific changes could be missed when analysing DNAm in blood, rather than tissues perceived to be more relevant to headache, such as brain. However, our observation that many of our differentially methylated genes are neuronally expressed and had high correlations between blood and brain DNAm, suggests that blood DNA can indeed reflect methylation changes in the brain. Furthermore, while our longitudinal study design greatly increases our power, direct replication of the findings in an independent cohort may be challenging given the intricacy of our clinical trial and longitudinal study design. Other designs, such as a large case–control study comparing DNAm between several hundred patients with chronic migraine and several hundred patients with episodic migraine, should be able to replicate DNAm associated with headache chronification. However, such cross-sectional studies will not be able to predict response for conversion to episodic migraine, as in the current study. Further work is required to understand the functional impacts of the implicated DNAm sites, such as their impact on gene expression. Lastly, our study only investigated DNAm and did not investigate other epigenetic changes, such as histone modifications.

Despite these limitations, our longitudinal study design is a powerful and robust method to detect within-individual DNAm changes caused by a response to acute medication treatment withdrawal. Other strengths include a well-characterised cohort of patients with chronic migraine and medication overuse, as opposed to studies including medication overuse headache, regardless of what underlying primary headache disorders might be present, and small cohort size.

## Conclusions

Understanding migraine chronification is vital for developing better treatment strategies and to prevent chronification. Epigenetic changes in genes represent potential treatment targets and identify mechanisms involved in migraine chronification. In summary, we identified a longitudinal reduction in *HDAC4* DNAm status associated with treatment response and implicated baseline *MARK3* DNAm status as an early biomarker for treatment response. Our findings provide support for a role of pathways related to chromatin structure, gene regulation, and synaptic plasticity in headache chronification and highlight *HDAC4* and *MARK3* as viable therapeutic targets, particularly considering convergence of their pathways and previous studies showing efficacy of general HDAC inhibitors in the treatment of migraine and MOH symptoms.

## Methods

### Study design and population

This study was conducted as part of the Chronification and Reversibility of Migraine (CHARM) clinical trial study at the Leiden Headache Center, which is described in detail elsewhere [4]. Briefly, consecutive patients aged 18–65 years, diagnosed with chronic migraine and medication overuse according to the International Classification of Headache Disorders (ICHD)-3 criteria [2], were enrolled. Exclusion criteria were: (i) other primary headache or neurological disorders; (ii) other chronic pain disorders with medium to high pain intensity or requiring pain medication; (iii) major psychiatric disorders, other than depression; (iv) major cognitive, behavioural or oncologic disorders; (v) contraindications for treatment, or inability to adhere to study protocol (vi) (planned) pregnancy or breastfeeding (vii) use of ergots, opioids or barbiturates; (viii) abuse of drugs in the past 12 months. The selected participants were a representative subset of the full CHARM cohort. No significant differences were found when selected participants were compared with non-selected participants on gender, BMI, MHD, MMD, and monthly medication days at baseline (*p* > 0.05). The selected participants were slightly older than the non-selected participants (mean ± SD: 47.0 ± 10.1 versus 43.1 ± 11.2, respectively, *p* = 0.02).

**Fig. 5:**
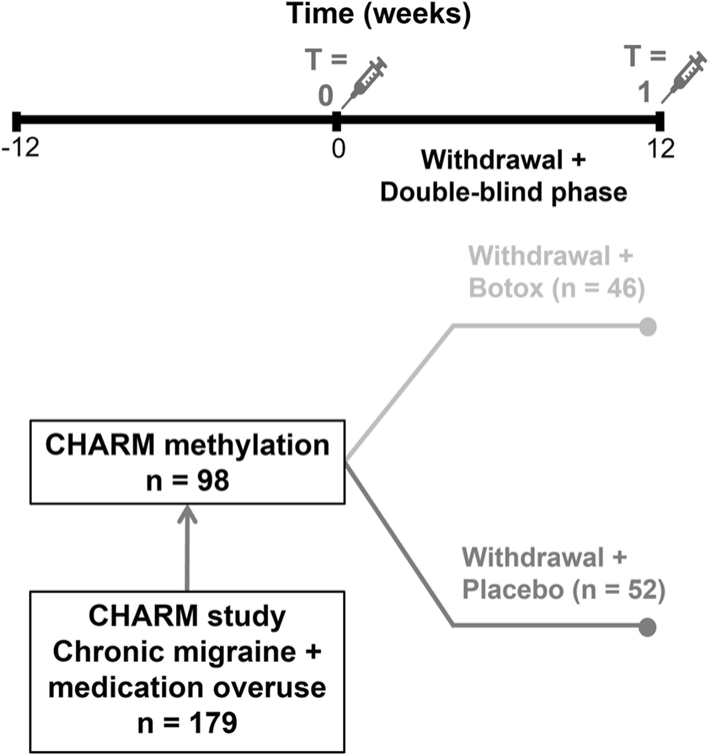
Study protocol. Botox = Botulin toxin A according to PREEMPT protocol, see Pijpers et al. (2019) [4]

Participants started with a 4-week baseline-assessment period followed by a 12-week withdrawal period, consisting of instruction to withdraw abruptly from all acute anti-headache medications and caffeine (‘advice-only’). Prophylactic treatment was tapered off and rescue medication was not allowed. Immediately prior to withdrawal, botulinum toxin A (botox) or placebo injections were administered in a randomised, double-blind manner. In the 12-week withdrawal period, no other prophylactics were started. Blood samples were taken at baseline (*T* = 0) and at 12 weeks (*T* = 1) (Fig. 5). Patients were selected from the CHARM study based on DNA sample availability, DNA concentration and order of participation. The sample size was well-powered to detect effect sizes equivalent to those found in previous longitudinal studies [48].

### Clinical outcomes

All participants prospectively kept a 4-week diary during the baseline assessment period and the post-treatment period (weeks 9–12). This included daily registration of headache characteristics, accompanying symptoms and acute headache medication used. A migraine day was defined as a day fulfilling criteria for migraine or treated with migraine-specific acute medication [2]. Headache days were defined as a day with a migraine or non-migraine headache. We compared changes in DNAm in monthly headache days (MHD) responders (defined as patients with ≥ 50% reduction in MHD) versus non-responders (patients with < 50% reduction in MHD), and in monthly migraine days (MMD) responders (defined as patients with ≥ 50% reduction in MMD) versus non-responders (patients with < 50% reduction in MMD). We also performed an exploratory analysis with the outcome of reversion from chronic to episodic migraine (i.e., headache no longer fulfilling criteria of chronic migraine).

### Samples

Genomic DNA was extracted from peripheral blood leukocytes according to standard protocols. A total of 196 samples were included for DNAm and analysed using the Illumina EPIC DNA methylation arrays (> 860,000 CpG sites at single-nucleotide resolution), using standard manufacturer protocols at the Genomics Research Centre, Queensland University of Technology. Briefly, 500 ng genomic DNA was converted with bisulfite treatment using EZ DNA Methylation Kits (Zymo Research, USA). Then, samples were whole-genome amplified, enzymatically fragmented, and hybridised overnight to locus-specific probes on Illumina Infinium EPIC Beadchips. After a wash step, hybridised probes on the Beadchips underwent a single-base extension reaction followed by a multilayered staining process before scanning on an Illumina HiScan for detection of probe intensities.

### Quality control and statistical analysis

The raw values from the Illumina EPIC DNA methylation arrays were imported using GenomeStudio software (Illumina) and uploaded into R (https://www.r-project.org/, version 3.4.0) for further processing. Data were analysed using an established analysis pipeline comprising custom statistical programs and scripts in R [49]. Samples with probe detection call rates < 95%, as well as those with an average intensity value of either < 50% of the experiment-wide sample mean or < 2,000 arbitrary units (AU), were excluded from further analysis. Intensity readouts, normalisation and DNAm beta value (the ratio of the fluorescent signals for the methylated versus unmethylated sites) calculations were performed using the Bioconductor MINFI package version 1.20.2 [50]. Subset-quantile Within-Array Normalisation (SWAN) was used to remove technical differences between Infinium I and Infinium II probes available in the MINFI package [51]. The DNAm status for each probe was recorded as a β-value that ranged between 0 and 1, where values close to 1 represent high levels of DNAm and values close to 0 represent low levels of DNAm. Probes with > 50% of the samples with a detection *p* value > 0.05 and probes with single nucleotide polymorphisms (SNPs) present within 50 bp from the query site were removed. This resulted in a total of 865,823 CpG probes for further analyses.

Using the MINFI package, initial quality checks were performed to predict the sex status of the samples and check for consistency with the true sample sex (predicted sex was consistent with true sex for all samples). Cell type composition was predicted using the DNAm data (CD8T, CD4T, NK, B-cell, Monocytes and Granulocytes) using the Houseman method [52].

Linear mixed effects models were used to investigate differential DNAm across all 865,823 CpG probes and test its association with phenotypes as repeated measures using the lme4 package in R. Details of the statistical analysis models used, including the R syntax of the analysis models, are provided in Additional file 3: Supplementary Methods. Applying these analysis models in our longitudinal study design is highly powerful, as they retain the within-person structure (repeated measures across a person) while assessing longitudinal differences in DNAm across the timepoints (before and after treatment). That is, one of the major sources of DNAm variability is between-participant variability. Therefore, by repeating measures within participants, each participant acts as its own control, and the between-participant variability is removed. Hence, our paired-test study design is more likely to detect true differences in DNAm between the paired measures and is a powerful approach. Indeed, the within-person Spearman correlations ranged between *r* = 0.959–0.989, with an average and standard deviation (*r*[SD]) = 0.979[0.009], this is much higher than that observed in MZ twins [53]. Even ignoring this increased correlation, our study design is at least equivalent to comparing 100 unrelated cases to 100 unrelated controls, for which power calculations using the EPIC array power calculator [54] found at https://epigenetics.essex.ac.uk/shiny/EPICDNAmPowerCalcs/, indicate 24.4% of CpG sites have > 80% power to detect an effect of 2%, 53% of sites have > 80% power to detect an effect of 3%, 72.8% of sites have > 80% power to detect an effect of 4%, and 85% of sites have > 80% power to detect an effect of 5%. These estimates of power are conservative given the longitudinal study design and the high within-person correlation we observe in the study. In summary, our study is well-powered to detect small to moderate DNAm differences. Furthermore, we evaluated whether DNAm status at baseline could be used to predict response to withdrawal using the glm function in R. All analyses were corrected for age, sex, smoking, botox injection, body mass index (BMI), and cell type counts.

We also performed post-hoc sensitivity analyses to account for prophylactic use and medication overuse type, and found all results remained significant after adjusting for medication use (*p* < 9.42 × 10^–8^). We previously demonstrated in the CHARM study patients with chronic migraine and medication overuse, that botox did not lead to any additional benefits over acute withdrawal alone [4]; however, to ensure that the observed results were not caused by botox injection, we controlled for botox treatment status in our primary analyses and also performed a post-hoc analysis to evaluate whether both the placebo and botox injection group demonstrated an association. None of the presented results were affected by botox injection. Top-ranked CpGs that met a stringent significance level of *p* < 9.42 × 10^–8^ were considered *significant* at the epigenome-wide level—the recommended threshold accepted to reduce the rate of false positives in DNAm studies [54]. The significance level of *p* < 5 × 10^–5^ was used to denote *suggestive* sites of relevance at the epigenome-wide level [55], and sites with *p* < 0.05 were considered nominally significant.

The Blood Brain DNA methylation comparison tool [56] was used to check the correlation between blood and brain DNAm for the significant CpGs. This tool was established for the older 450 K DNA methylation array and therefore does not contain all the CpG probes analysed in this study. To compare results to known genes associated with migraine and/or headache traits, we compiled a list of 184 genes (using the closest gene to a genome-wide significant SNP) identified from five GWASs for migraine and headache [20–24], of which 118 unique genes corresponding to 4,386 CpGs were present in the current dataset. To test whether the overlap of genes was more than expected by chance, enrichment testing was performed using 1,000 permutations (using random sets) and applying a two-sided Binominal test in R to give a *p* value of enrichment. Functional annotation of the genes corresponding to the CpG sites at the suggestive genome-wide significance level (*p* < 5 × 10^–5^) was performed and the Drug-Gene Interaction database (DGIdb, https://www.dgidb.org/, v4.2.0) [57] was used to assess whether these sites were in genes involved in drug-gene interactions—i.e., whether there is a known interaction (e.g., inhibition) between a known drug and a target gene. These genes are targeted by specific known compounds (i.e., it describes whether the gene is ‘currently actionable’). We also assessed whether genes were druggable candidates according to the DGIdb. Druggable candidates are genes that are thought to be potentially druggable by various methods of prediction. As such, genes in these categories are ‘potentially druggable’ and may or may not have existing drugs that target them.

### Supplementary Information

**Additional file 1**. **Figure S1.** Q-Q plot of the p-values from the epigenome‐wide association study of changes in DNA methylation in monthly headache days (MHD) responders versus non-responders.

**Additional file 2**. **Table S1.** CpGs nominally associated with MHD response following withdrawel of acute medication located at the *HDAC4* locus (*p* < 0.05). **Table S2.** CpGs associated with MHD response following withdrawel of acute medication at the suggestive level of significance (*p* < 5 × 10^-5^). **Table S3.** CpGs associated with MMD response following withdrawel of acute medication at the suggestive level of significance (*p* < 5 × 10^-5^). **Table S4.** CpGs associated with conversion from CM to EM following withdrawel of acute medication at the suggestive level of significance (*p* < 5 × 10^-5^). **Table S5**. CpGs at baseline associated with MMD response at T1 at the suggestive level of significance (*p* < 5 × 10^-5^). **Table S6.** CpGs at baseline associated with MHD response at T1 at the suggestive level of significance (*p* < 5 × 10^-5^). 

**Additional file 3**. **Supplementary Methods.** The R syntax of the longitudinal linear mixed effects analysis model and the baseline glm analysis model.

## Data Availability

The data that support the findings of this study are available from the corresponding authors, upon reasonable request.

## Abbreviations

2-AG: 2-Arachidonylglycerol
AMPA: α-Amino-3-hydroxy-5-methyl-4-isoxazolepropionic acid
AU: Arbitrary units
CHARM: Chronification and Reversibility of Migraine
CpG: 5′-Cytosine-phosphate-guanine-3′
CSD: Cortical spreading depolarisation
DAT: Dopamine transporter
DGIdb: Drug-Gene Interaction database
EM: Episodic migraine
EWAS: Epigenome-wide association study
GWAS: Genome-wide association study
HDAC4: Histone deacetylase 4
ICHD: International Classification of Headache Disorders
MHD: Monthly headache days
MMD: Monthly migraine days
NMDAR: *N*-Methyl-d-aspartate receptor
SNP: Single nucleotide polymorphism
SWAN: Subset-quantile Within-Array Normalisation
TSS: Transcriptional start site
