# Supplementary material for: Alterations in DNA methylation associate with reduced migraine and headache days after medication withdrawal treatment in chronic migraine patients: a longitudinal study
Source: Clin Epigenetics. 2023 Dec 12;15:190. doi: 10.1186/s13148-023-01604-8 (PMC10717674; doi:10.1186/s13148-023-01604-8)
Supplement: Supplementary file 1 — Additional file 1. Figure S1. Q-Q plot of the p-values from the epigenome‐wide association study of changes in DNA methylation in monthly headache days (MHD) responders versus non-responders. [file 13148_2023_1604_MOESM1_ESM.docx]

**
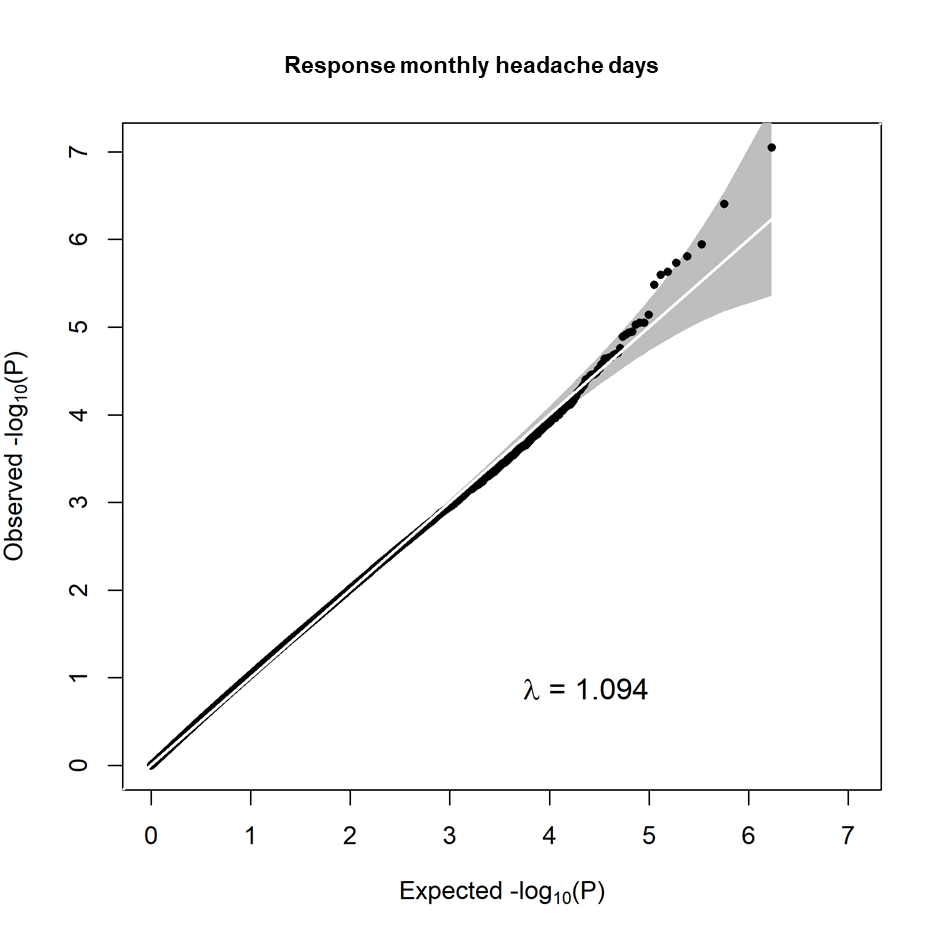
**

**Fig. S1** Q-Q plot of the p-values from the epigenome‐wide association study of changes in DNA methylation in monthly headache days (MHD) responders versus non-responders (black data points). The plot was constructed by ranking p-values from smallest to largest (the 'order' statistics) and plotting them against their expected values under the null hypothesis of no association (samples from the known chi-squared distribution). Deviations above the line of equality (drawn in white) indicate a preponderance of smaller p-values. To aid interpretation, we have also calculated 95% confidence envelopes (shaded grey). These are formed by calculating, for reach order statistic, the 2.5th and 97.5th centiles of the distribution of the order statistic under random sampling and the null hypothesis. The genomic inflation factor (lambda, λ) is also shown, defined as the ratio of the median of the empirically observed distribution of the test statistic to the expected median, thus quantifying the extent of the bulk inflation. The Q-Q pot and low λ (close to 1) indicate that these test statistics data have no systematic technical bias or inflation.
