# Supplementary material for: Alterations in DNA methylation associate with reduced migraine and headache days after medication withdrawal treatment in chronic migraine patients: a longitudinal study
Source: Clin Epigenetics. 2023 Dec 12;15:190. doi: 10.1186/s13148-023-01604-8 (PMC10717674; doi:10.1186/s13148-023-01604-8)
Supplement: Supplementary file 3 — Additional file 3. Supplementary Methods. The R syntax of the longitudinal linear mixed effects analysis model and the baseline glm analysis model. [file 13148_2023_1604_MOESM3_ESM.docx]

**Supplementary Methods**

**The R syntax of the longitudinal linear mixed effects analysis model is:**

mod1 <- lmer(DNAm ~ response * timepoint + (1 | patient) + age + sex + smoking + botox + BMI + CD8T + CD4T + NK + Bcell + Monocytes + Granulocytes)

mod2 <- <- lmer(DNAm ~ response + timepoint + (1 | patient) + age + sex + smoking + botox + BMI + CD8T + CD4T + NK + Bcell + Monocytes + Granulocytes)

lm <- anova(mod1, mod2)

where response is MHD or MMD, and (1 | patient) is the random effect modelling the repeated measures in each patient.

NB: response * timepoint = response + timepoint + response:timepoint

Therefore, lm <- anova(mod1, mod2) provides the *p* value of the response:timepoint interaction term and thus tests for an association between DNAm level and change in response MHD or MMD over time.

**The R syntax of the baseline glm analysis model is:**

mod3 <- glm(DNAm ~ response + age + sex + smoking + botox + BMI + CD8T + CD4T + NK + Bcell + Monocytes + Granulocytes)

where response is the change in MHD or MMD from T0 to T1.
